# The Liquid Limit as a Factor in Assessing the Improvement of Stabilized Cement-Based Highwater Content Clayey Sediments

**DOI:** 10.3390/ma15207240

**Published:** 2022-10-17

**Authors:** Zichen Zhang, Kiyoshi Omine, Samuel Oye Flemmy, Cui Li

**Affiliations:** Graduate School of Engineering, University of Nagasaki, Nagasaki 8528521, Japan

**Keywords:** high water content, clayey sediment, cement, fly ash, liquid limit, improvement

## Abstract

The purpose of this study was to assess the performance of high water content clayey sediments at different liquid limits as the clays are treated with cement-based solidifying materials. Three clay samples are obtained from different locations in the Kumamoto Reservoir. Two types of cement-based solidifying agents, namely, ordinary Portland cement and a cement–fly ash binder, were used. Using the initial water content of clay and the mixing amount of the solidifying agent as experimental variables, a cone penetration test was performed on the solidifying agent-stabilized clays to obtain the cone index (*q*_c_). The results showed that when the water content to cementitious content ratio (*w*/*A*_W_) was used as a parameter for evaluating the improvement of solidifying agent-stabilized clay, different forms of improvements were observed when different water and solidifying agent contents were used. This implied that the parameter *w*/*A*_W_ was not suitable for evaluating the improvement of such clay. A new parameter, *K*, representing the content of solidifying agent, was introduced to account for the water content. For all sampled clays, the correlation coefficients for the *K*–ln *q*_c_ relationship exceeded 0.9. Considering the effect of the liquid limit of the samples, the modified content of the solidifying agent (*K*_L_) was introduced to evaluate the cone index of the stabilized soils. It was discovered that the proposed equation unified the assessment of the improvement of the three samples of Kumamoto clayey sediments owing to the new parameter, *K*_L_.

## 1. Introduction

The construction and renovation of infrastructures such as reservoirs and swamps necessitate the treatment of a significant amount of clay at the bottom. According to the Ministry of the Environmental Survey in Japan, there are more than 100,000 reservoirs and artificial lakes, exceeding 10 million cubic meters [1]. From 1978 to 2010, owing to the decline in the water level line, sediment accumulation in reservoirs increased annually [2]. The disposal of these undesired liquid clays poses significant challenges. Reservoir clay has a high water content, high compressibility, and low shear strength. Therefore, the mechanical properties of the clay must be improved before it is used for construction. Clay improved using cement-based solidifying agents has been extensively used in highway and port construction in many countries, such as Japan, Thailand, and Nigeria [3,4,5,6].

Cement has been widely used to improve soft clay content. At the initial stage of mixing the clay cement, the natural bonds inside the clay matrices are destroyed, whereas new bonds are created owing to the hydration reaction of cement. Despite the advantage of ordinary Portland cement (OPC) in soil improvement, several disadvantages have been identified, such as the significant amount of carbon dioxide emission during cement production. Fly ash is one of the materials extracted from the flue gases of a furnace fried with coal from an electric power plant. Its generation is significantly greater than its utilization. In fact, researchers have attempted to use fly ash from industries to reduce cement input [7,8,9,10]. Fly ash is generally composed of silica, alumina, and ferric oxides. These compounds form cementitious materials by combining with cement in the presence of water [11]. Fly ash is a supplementary cementing material in concrete mixtures and is used as a partial substitute in concrete cement [12].

A simple method has been established for soft clay admixed with cement to estimate the unconfined compressive strength (*q*_u_) of cement-treated soils while considering cement and water contents [13]. The clay–water/cement ratio (*w*/*C*), which is the ratio of the initial water content of the clay to the cement content, is a standard parameter for investigating the engineering behavior of cement-stabilized clays at high water contents [14]. The generalized strength equation based on Abrams’ law [15] can be realized as the target strength and the desired levels of strength and compressibility obtained in laboratory tests [16]. It was demonstrated that *C*/*w* is an effective parameter for predicting the improvement of cement-stabilized clays [17]. The cement content required to stabilize roof tile waste silt soil can be estimated using strength equations involving the unconfined compressive strength and porosity/volumetric cement content ratio [18]. Many studies have focused on the behavior of cemented clay. However, the improvement afforded by cement-based solidifying materials is significantly affected by the unique physical properties of the clay, such as its particle size composition, organic matter (OM), clay minerals, and pH [19,20]. Therefore, for reservoir clay with high organic matter content and high natural water content, the applicability of the evaluation behavior should be considered.

Clayey sediments typically exhibit high water and organic matter contents, which vary widely based on region. Therefore, the improvement by solidifying agents should be assessed under different mixing conditions. In this study, Kumamoto clayey sediment was obtained from three locations in the Kumamoto reservoir. OPC and cement–fly ash binder were used for the improvement. The improvement of the three types of clayey sediments with high water content was expounded in terms of the solidifying agent and water contents. A cone penetration test was performed on solidifying agent-stabilized clays to obtain the cone index. An empirical equation for assessing the improvement of Kumamoto clayey sediments mixed with cement-based solidifying agents is discussed herein based on laboratory test results and a new parameter, which is the modified content of the solidifying agent.

## 2. Test Procedure

### 2.1. Characterization of the Clayey Sediment

**Figure 1:**
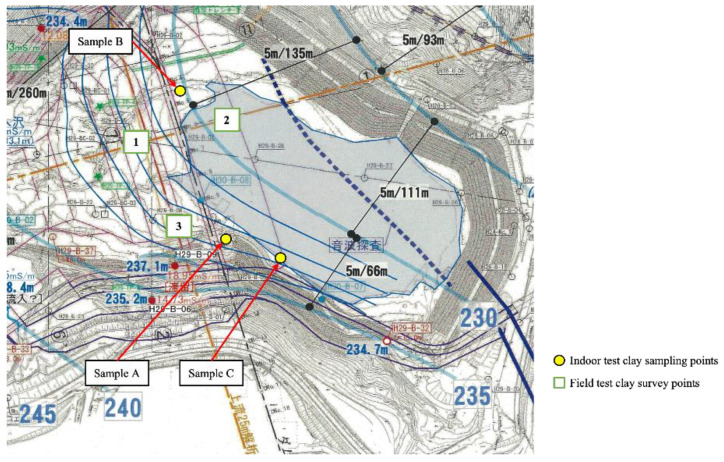
Locations where dynamic cone penetration test and water content measurement were performed; sampling position of Kumamoto clay.

As shown in the reservoir plan presented in Figure 1, to confirm the depth of the clay sediment in the Kumamoto reservoir, a dynamic cone penetration test [21] was performed in areas № 1 to 3. The driving hammer with a mass of 5 kg was freely dropped from a height of 0.5 m, and the cone penetrometer had an initial angle of 60° and a diameter of 25 mm and the number of blows per penetration depth of 0.1 m (*N*_d_) required to penetrate 0.1–0.5 m cone was measured. To determine the linear relationship between *N*_d_ and water content, the water content was measured for soil samples at a depth of 0.1–0.5 m. A loss -on-ignition test was performed at 750 °C [22] on the clays obtained from three locations to determine the organic matter content.

**Figure 2:**
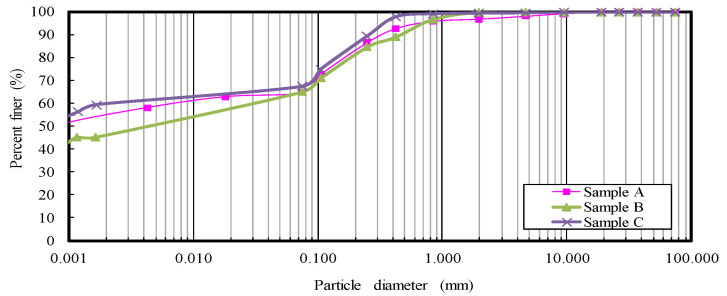
Grain size distribution of Kumamoto clay.

Two types of cement-based solidifying agents were used. One was type 1 OPC, and the other was a cement–fly ash binder (DF). DF comprises OPC (30%), coal fly ash (58%), and a heavy metal dissolution inhibitor (12%). Heavy metal dissolution inhibitors are composed of inorganic metals and chemicals. By mixing with heavy metal dissolution inhibitors, the effect of inhibiting the dissolution of natural heavy metals in soil was achieved. To clarify the effect of the physical properties of clayey sediment on the improvement, three different locations (in Figure 1) were selected to obtain clay samples (samples A, B, and C). The physical properties of clayey sediment are listed in Table 1. The grain size distribution of clayey sediment obtained from the particle size test [23] is shown in Figure 2. The test results show that the fine particle content of all the samples constituted more than 60% of the clay. The water content of the three types of samples in the natural state was 160% in sample A, 152% in sample B, and 208% in sample C. Based on the experimental results of the liquid and plastic limit of each sediment sample [24], the water content of the three samples in the natural state is 10% to 50% higher than the liquid limit. Based on the results of the loss-on-ignition (LOI) test performed at 750 °C, the range of LOI in the sediments was between 17% and 24%. A uniaxial compression test [25] was performed on an undisturbed sample obtained from the location where sample C was acquired. In addition, the sample was kneaded after the uniaxial compression test, and a uniaxial compression test was performed to obtain the sensitivity ratio.

### 2.2. Sample Preparation

All the clay was passed through a 4.75 mm sieve to remove the coarser particles. In the water content adjustment experiments, the water content value was derived from a water content test [26]. The water content of the clay was adjusted by adding free water and placing it in the natural air-drying location at the ventilating place, and the water content of all the experimental clays was equal to the target water content. During specimen preparation, the target clay was first mixed uniformly with a mortar mixer. Then, the solidifying agent was added to the clay and fully mixed until the whole sample attained form consistency. In order to prevent evaporation and absorption of moisture in the test sample, the test sample was sealed with macromolecular polyethylene, and then the sealed test sample was placed under constant temperature (25 ± 3 °C) and humidity (90 ± 3%). Sample preparation was based on the standard of the Japanese Geotechnical Society’s method of preparing samples by ‘Practice for making and curing compacted stabilized soil specimens using a rammer’ [27]. Table 2 shows the cone penetration test conditions. After 28 days of storage, the sample was compacted in a mold with a height of 127 mm and a diameter of 100 mm using a hammer with a mass of 2.5 kg and a drop height of 300 mm. A compaction layer was set at about 40 mm intervals to press the sample into the mold three times, and each layer had 25 compaction cycles (JIS A 1210 A-c method: compaction energy about 550 kJ/m^3^) [28]. Then, a cone penetration test was immediately performed.

### 2.3. Cone Index Determination

A cone penetration test was performed on the solidifying agent-stabilized clays to determine the cone index [29]. Table 3 summarizes the experimental conditions for the improved clay. Two series of experiments were conducted in this study using different solidifying agent contents (*C*) and initial water contents (*w*). In the first series of experiments, the initial water content of the clay was constant, i.e., the initial water content of samples A and B was 160%, and that of sample C was 200% because of the higher liquid limit. Different contents of solidifying agents DF and OPC were used: 100, 200, 300, and 400 kg/m^3^. The objective of series 1 was to investigate the effect of the solidifying agent content on the cone index of the improved clays. For series 2, the content of the solidifying agent was constant, i.e., 100 kg/m^3^ for all the samples. The initial water content of the clay was different for each clay. The aim of series 2 was to investigate the effect of the initial water content on the improved clays. The penetration resistance of the test sample at 50, 75, and 100 mm was measured, and the average value divided by the cone bottom area was defined as the cone index. For each clay type, solidifying agent type, and combination of water content and cement content, at least three samples were tested under the same conditions to check for test consistency. In most cases, the results under the same testing condition were reproducible.

## 3. Results

### 3.1. On-Site Sediment Characterization

**Figure 3:**
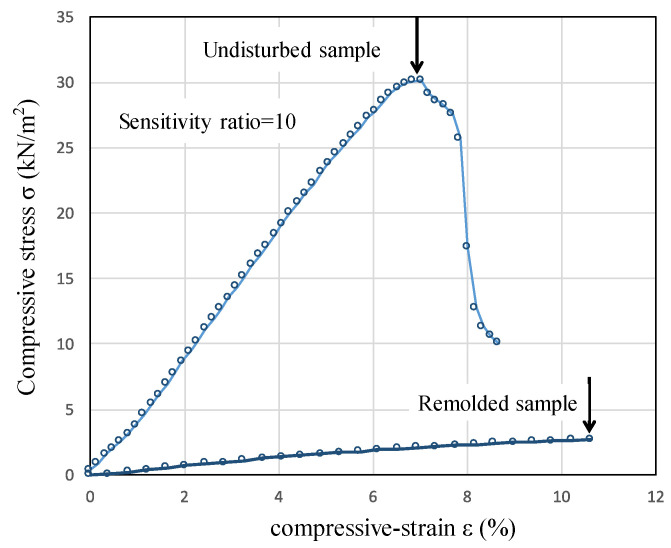
Uniaxial compression test results of undisturbed and remolded clays of sample C.

The uniaxial compression test results for the undisturbed and remolded clays of sample C are shown in Figure 3. The sensitivity ratio (*S*t) is defined as the compressive strength (*q*_u1_) of undisturbed clays relative to the compressive strength (*q*_u2_) of remolded clays. Clays with a value of *S*t exceeding 10 were classified as super-sensitive clay. The test results indicated an *S*t = 11; therefore, Kumamoto sediment was regarded as a super-sensitive clay. This means that although the Kumamoto sediment exhibited 30 kPa of unconfined compressive strength, its strength would become very low, less than 3 kPa, once disturbed.

When burned at high temperatures, the organic matter in clay begins to ignite at about 200 °C and will be completely depleted at about 550 °C [30]. Loss-on-ignition (LOI) is a parameter that represents the organic matter content [31]. Generally, clays with an LOI of more than 20% are regarded as highly organic clay [32]. The LOI test was carried out on the sediments from areas № 1 to № 3. The results showed that LOI = 26.15% in area № 1, and that of the samples in areas № 2 and 3 exceeded 30%. In addition, combined with the LOI test results of samples A–C in Table 1, the sediment was classified as high-organic clay.

**Figure 4:**
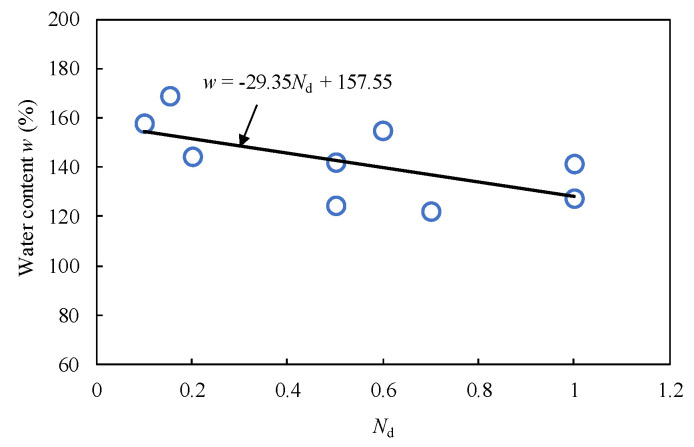
Relationship between water content and *N*_d_ obtained from dynamic cone penetration test.

Figure 4 shows the relationship between the water content and the number of blows per penetration depth of 0.1 m (*N*_d_) determined by the dynamic cone penetration test. The sediment samples for measuring the water content were obtained from a depth range of 0.1 to 0.5 m from the surface of the Kumamoto reservoir. Based on the results of all 3 samples, the following correlations were derived:

*w* (%) = 157.55 − 29.35*N*_d_ (1)

**Figure 5:**
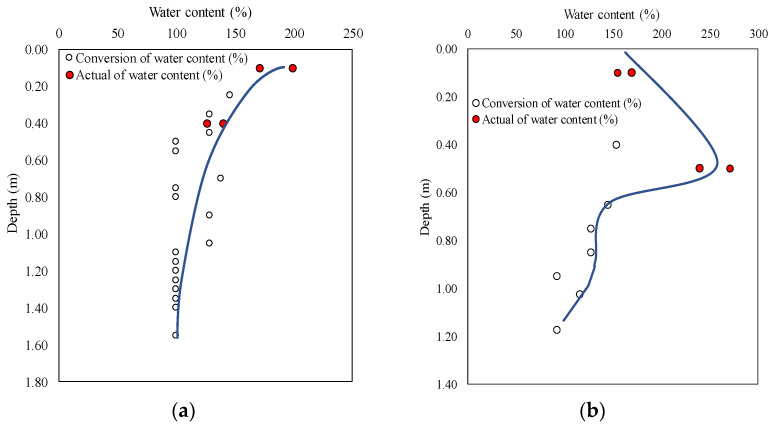

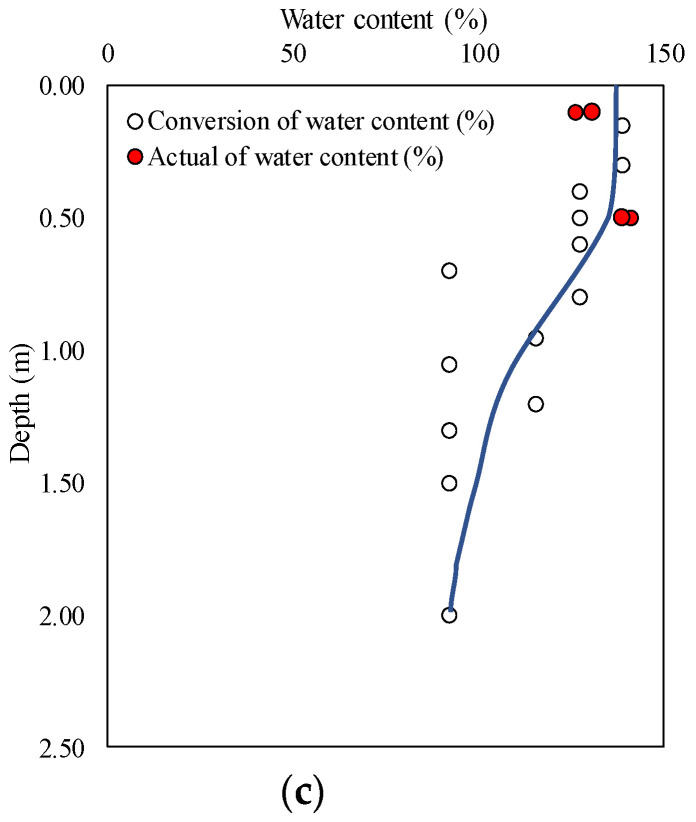
Change in water content based on depth in areas № 1–3. (**a**) area № 1, (**b**) area № 2, (**c**) area № 3.

As shown in Equation (1), the water content in the depth direction can be calculated using *N*_d_. Figure 5 shows the distribution of converted water content with depth in the areas № 1–3 calculated from *N*_d_. The purpose of measuring the actual water content is to correct the distribution curve. This figure shows the distribution of natural water content in the sediments of the Kumamoto reservoir. It was discovered that clay with a high water content (exceeding 100%) was deposited at a depth of 1 to 2 m.

### 3.2. Effect of OPC and Cement–Fly Ash Binder on the Clayey Sediment Improvement

In soil stabilization by cement, cement hydration provided calcium silicate hydrate (CSH) and calcium aluminate hydrate (CAH), resulting in increased improvement at a curing period of 28 days [33]. Similar to OPC, DF indicated increased strength owing to the reaction of calcium hydroxide with siliceous and aluminous materials to produce CSH and CAH [34]. The original water content in the clay promoted hydration and pozzolanic reactions, which rapidly generated ettringite. The hydration products of the cement were observed in the pores of the microstructure, and the amount of cementitious products increased significantly. The cementitious products not only enhanced the inter-cluster bonding strength, but also filled the pore space. The volume of pores smaller than 0.1 μm reduced significantly, thereby reducing the total pore volume. Consequently, the strength increased significantly [35]. The fly ash particles were discovered to be beneficial for reducing voids in the clay structure and making the microstructure denser, resulting in improved clay strength after curing [36,37].

**Figure 6:**
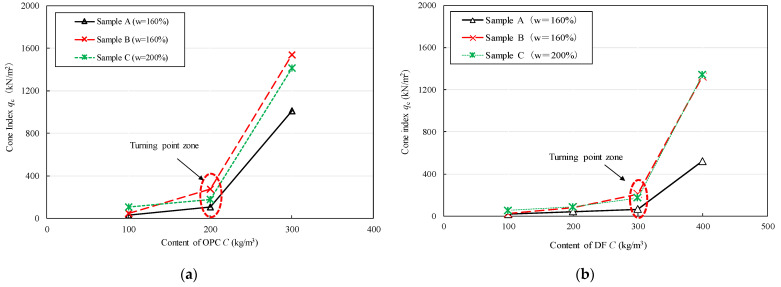
Relationship between cone index of stabilized clay and content of solidifying agents. (**a**) Stabilized clay by OPC, (**b**) Stabilized clay by DF.

In series 1 under the constant water content condition (samples A and B: *w* = 160%; sample C: *w* = 200%), the relationship between the content of the solidifying agent and the cone index is shown in Figure 6a,b. As shown in Figure 6a, the OPC reached the improved ‘turning point zone’ when the content reached 200 kg/m^3^. This means that more than 200 kg/m^3^ of stable clay had been significantly improved. Meanwhile, the cone index of the soil stabilized by DF increased rapidly when the content of DF exceeded 300 kg/m^3^, as shown in Figure 6b. When using cement–fly ash binder containing 30% cement to stabilize sediment with high water content, a certain amount of hydration products must be produced to facilitate the embedding of fly ash particles into the gap of gel materials. Therefore, the DF needs more mixing quantity to reach the ‘turning point zone’ in the improved Figure 6b. It was assumed that DF was applicable for improving Kumamoto clay, although the required content of DF was much higher than that of OPC. Since the OPC content in DF was only 30%, it afforded low CO_2_ emission in comparison with OPC.

### 3.3. Effect of Reducing Water Content of Clayey Sediment

**Figure 7:**
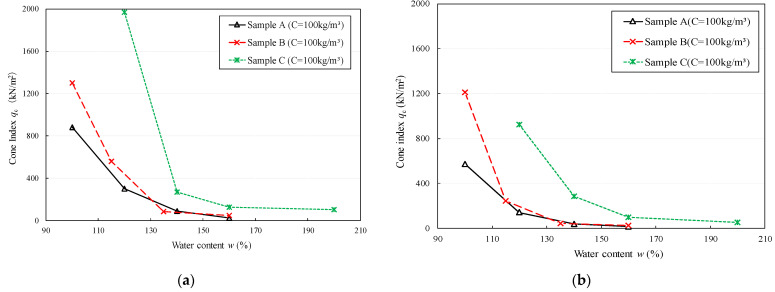
Relationship between cone index and water content at constant content of solidifying agent (100 kg/m^3^). (**a**) Stabilized clay by OPC, (**b**) Stabilized clay by DF.

For the test results of series 2, Figure 7a,b shows the relationships between the cone index and the initial water content of the three types of Kumamoto clay under a constant content of the solidifying material (100 kg/m^3^). The cone index of the soils stabilized by the solidifying agents significantly decreased as the initial water content increased. This is because the sample with a higher clay water contents is easy to break up the cementation bond under the influence of higher void ratios, which affects the hardening rate [14]. Consequently, the improvement became less prominent as the water content increased. By contrast, the decrease in the water content of the clay reduced the distance between the particles or cluster of particles, and the amount of hydration products increased. Under the same improvement conditions, the degree of improvement depended on the OM content of the sediment. The higher the OM content, the more difficult it was to effectively improve. 

## 4. Discussion

### 4.1. The w/A_w_ Ratio as a Factor for Assessing the Improvement of Stabilized Clayey Sediment

It is important to predict the improvement of stabilized clays for preliminary design and cost analysis. In previous studies, the contents of stabilization binders and water have been proven to be the main factors affecting improvement [38,39,40]. Some researchers defined the content of stabilized binder (*A*_w_) as the ratio of stabilization binder weight to the dry weight of clay and used the ratio of water content (*w*) to *A*_w_ as a controlled parameter to study the behavior of stabilized clay. The results show that *w*/*A*_w_ described well the mechanical properties of stabilized clay, and the correlation coefficient R^2^ reached 0.953 [41,42]. In this section, an assessment of the improvement by the solidifying agents is provided while considering the effect of *w*/*A*_w_.

**Figure 8:**
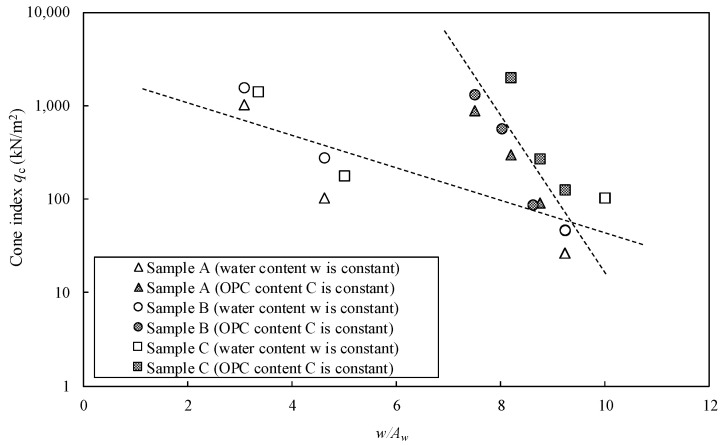
Cone index (*q*_c_) vs. *w*/*A*_w_ ratio of clays stabilized by OPC in three types of Kumamoto clay.

**Figure 9:**
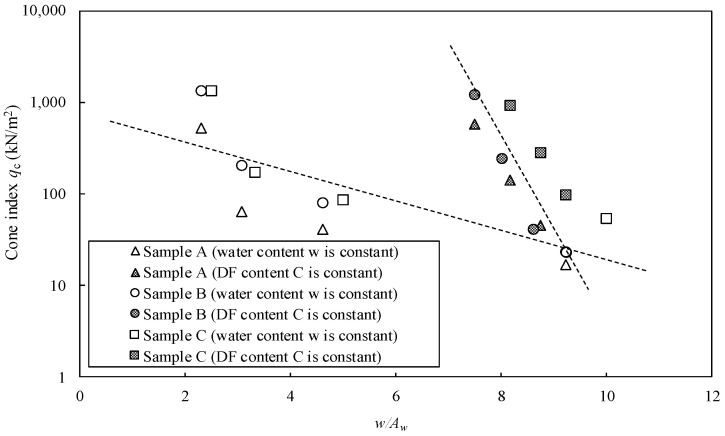
Cone index *q*_c_ vs. *w*/*A*_w_ ratio on the stabilized clays by DF in three types of Kumamoto clay.

Figure 8 shows the relationship between the cone index (*q*_c_) and *w*/*A*_w_ on the clay stabilized by OPC in the three types of Kumamoto clay. The results indicate different trends under constant water and solidification agent contents. The difference in *q*_c_ became more evident as *w*/*A*_w_ decreased. A similar trend was observed for the clay stabilized by DF, as shown in Figure 9. All *q*_c_–*w*/*A*_w_ relations did not show a unique line; therefore, it was concluded that the previous evaluation method was not suitable for Kumamoto clay with high water and organic matter contents.

### 4.2. The K Parameter as a New Factor for Assessing the Improvement of Stabilized Clayey Sediment

Highly organic soil contains bitumen, fulvic acid, and other humic acids with different degrees of organic matter decomposition. Substances in the humic acid dissolved in acidic methanol hydrochloride affect the solidified strength [43]. In addition, the inhibition of cement hydration by humic acid occurs when eluted Ca^2+^ reacts with humic acid. Its calcium salt is deposited on the surface of unhydrated cement particles or forms a complex. Humic acid inhibits cement hydration and delays the reaction [44]. Therefore, for clays with high liquid limits and organic matter contents, a parameter to replace *w*/*A*_w_ should be considered. Considering the effect of the water content on *q*_c_, a new parameter, *K* (kg/m^3^), was introduced, as shown in Equation (2):

*K* = *C*/(*w*/100)*^d^* (2)

where *d* is a coefficient representing the effect of the water content, *C* (kg/m^3^) is the mixing content of solidifying agent, and *w* (%) is the water content of the clay. In this study, the following equation correlating *q*_c_ and *K* is used:

*q*_c_ = *A*exp(*BK*) (3)

where *A* (kN/m^2^) and *B* (m^3^/kg) are constants. Equation (3) can be converted into Equation (4), which shows a linear relationship between ln *q*_c_ and *K*.

ln *q*_c_ = ln*A* + *BK* (4)

**Figure 10:**
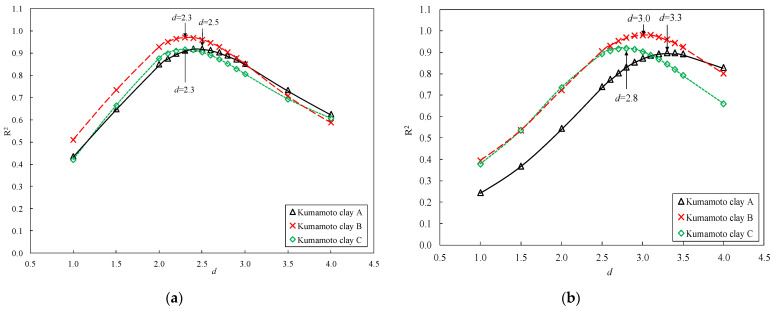
Change in correlation coefficient R^2^ of Equation (4) based on different *d* of clay stabilized by OPC or DF. (**a**) Stabilized clay by OPC, (**b**) Stabilized clay by DF.

Figure 10a,b shows the changes in the correlation coefficient (R^2^) of Equation (4) for different *d* values of the clay stabilized by OPC or DF, respectively. It was discovered that R^2^ indicated a peak value above 0.9 in all stabilized clays. In the clay stabilized by OPC, when R^2^ reached its peak, the corresponding *d* ranged from 2.3 to 2.5. In the clay stabilized by DF, the correlation coefficient (R^2^) reached its peak when *d* was 2.8–3.3. The value of *d* corresponding to the highest R^2^ was applied for each stabilized clay.

**Figure 11:**
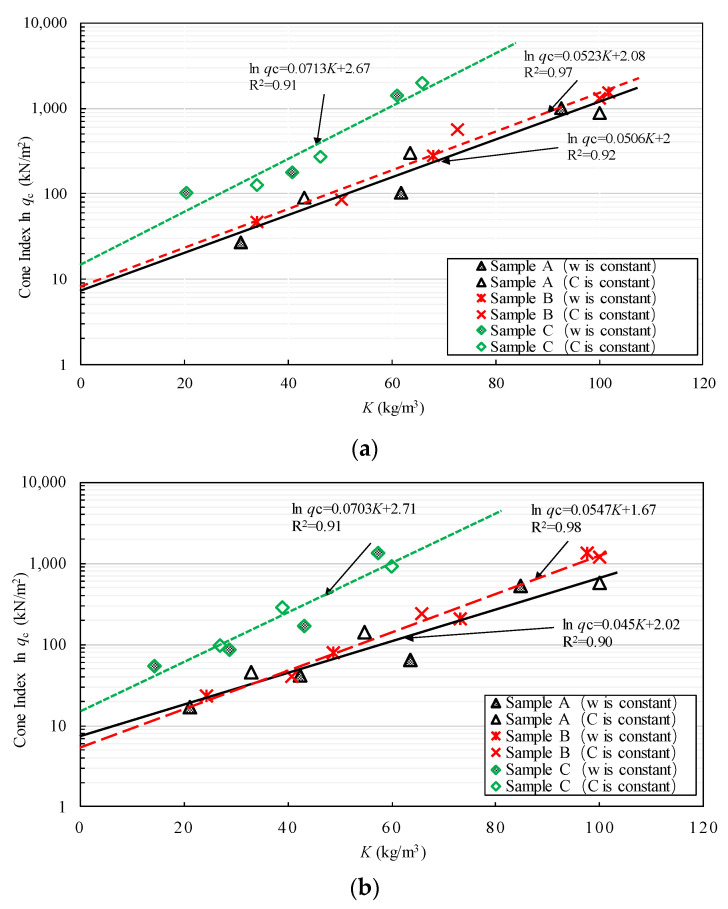
Relationship between ln *q*_c_ and *K* for clay stabilized by OPC or DF. (**a**) Stabilized clay by OPC, (**b**) Stabilized clay by DF.

Figure 11a,b shows the relationship between *q*_c_ and *K* obtained from the test results. The relationship between ln *q*_c_ and *K* can be represented by a straight line with an R^2^ exceeding 0.9 for all samples of Kumamoto clay. The constant *A* in Equation (4) represents the intercept of this line and corresponds to the value of *q*_c_ at *K* = 0, namely, the *q*_c_ of the clay without a solidifying agent. The constant *B* represents the inclination of the straight line. It is assumed that *B* and *d* are affected by the physical properties, such as the liquid limit and organic matter content of the clays. Table 4 summarizes the parameters *A*, *B*, and *d* for the stabilized clay obtained from Figure 11a,b. The improvement of each sample was assessed based on Equation (4). In other words, the proposed equation is crucial for calculating the required content of solidifying agents in an arbitrary water content in the clay to obtain the target cone index.

### 4.3. A Liquid Limit-Based Parameter as a Unified Factor for Assessing the Improvement of Stabilized Clayey Sediment

Although the expression shown in Equation (4) can estimate the cone index of each stabilized clay effectively, the effect of the physical properties of clay must be considered when assessing the improvement in a unified manner. Hence, a unified estimation formula must be devised to describe the improvement of all samples of Kumamoto clay. The *w*/*w*_L_ ratio is an important parameter for assessing the engineering properties of remolded and natural clays, where *w* is the water content of clay, and *w*_L_ is the liquid limit [45]. Considering the effect of the liquid limit of the samples, the content of the solidifying agent, *K* (kg/m^3^) (as indicated in Equation (2)), changes to the modified content of the solidifying agent *K*_L_ (kg/m^3^), as follows:

*K*_L_ = *C*/(*w*/*w*_L_)(*d*_L_) (5)

where *d*_L_ is a coefficient representing the effect of the water content based on the liquid limit. By introducing the new parameter, *K*_L_, Equations (3) and (4) can be redefined as follows:

*q*_c_ = *A*_L_ exp(*B*_L_*K*_L_) (6)

ln *q*_c_ = ln*A*_L_ + *B*_L_*K*_L_ (7)

where *A*_L_ (kN/m^2^) and *B*_L_ (m^3^/kg) are constants.

**Figure 12:**
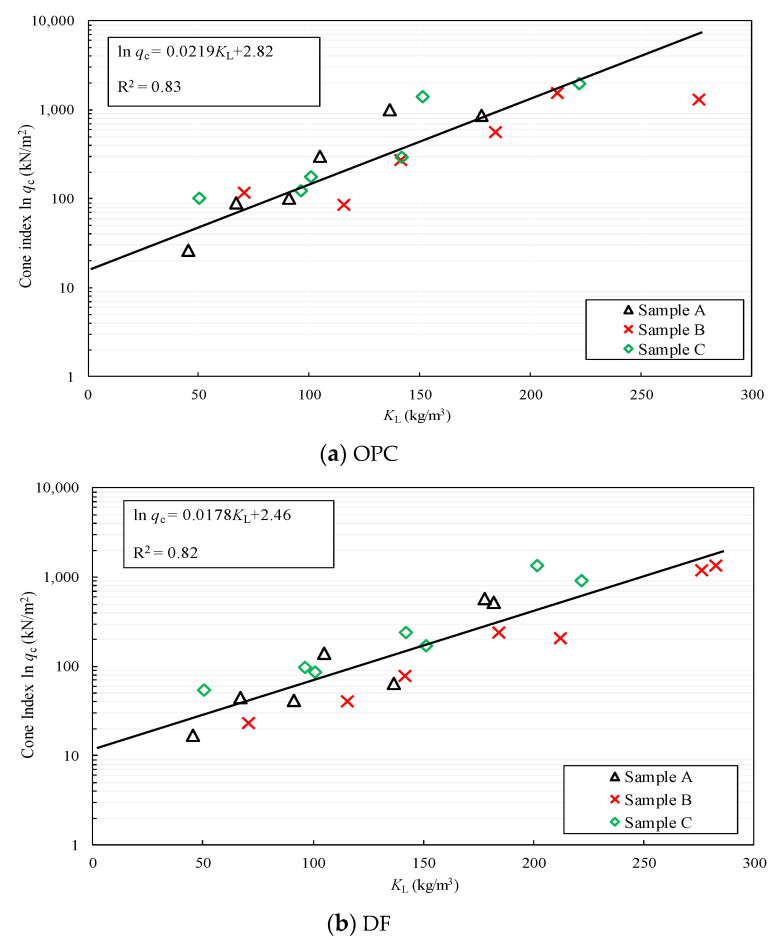
Relationship between ln *q*_c_ and *K*_L_ of clay stabilized by OPC or DF. (**a**) Stabilized clay by OPC, (**b**) Stabilized clay by DF.

Figure 12a,b show the relationship between *q*_c_ and *K*_L_ for the clays stabilized by OPC or DF. A straight line representing Equation (7) is presented in the figure in addition with the measured cone index of all the stabilized clays. It was observed that Equation (7) unifies the assessment of the improvement of Kumamoto clay using *K*_L_. Table 5 summarizes the coefficient *d*_L_ and the corresponding parameters *A*_L_ and *B*_L_, based on the highest correlation coefficient R^2^. The R^2^ of Equation (7) for the clays stabilized by OPC and DF were 0.83 and 0.82, respectively, which indicated good correlations and satisfied the number of data required for the significance level of 5%. As listed in Table 5, the value of *d*_L_ of the clay stabilized by DF was larger than that stabilized by OPC. This indicates that the improvement of the clay stabilized by DF was more sensitive to the water content compared with that stabilized by OPC. However, this suggests that the cone index of the stabilized clay with a higher value of *d*_L_ can be effectively increased by reducing the water content to less than the liquid limit. Based on this result, the authors attempted to reduce the water content of Kumamoto clay to less than the liquid limit using a simple dehydration method [46,47].

## 5. Conclusions

In this study, sediments from the Kumamoto reservoir were investigated via a field test. The experimental results indicated the presence of mass sediments of clay with high water and high organic matters within 1–2 m of the reservoir sediment. Two types of cement-based solidifying agents, i.e., OPC and DF, were added to three samples obtained from different locations, and a cone penetration test was performed. The following conclusions were obtained:

(1) DF was applicable to the improvement of Kumamoto clay, although the required content was much larger than that of OPC. DF is a recycling material composed of primarily fly ash; hence, it affords less environmental pollution owing to its low CO_2_ emission.
(2) The *w*/*A*_w_ was not applicable to Kumamoto clay with high water and organic contents as a parameter for evaluating the improvement of clay stabilized by OPC or DF.
(3) A new parameter representing the content of the solidifying agent, *K*, was introduced by considering the effect of water content. The relationship between ln *q*_c_ and *K* can be represented by a straight line with an R^2^ exceeding 0.9 for each sample of Kumamoto clay.
(4) Considering the effect of the liquid limit of the samples, the modified content of the solidifying agent, *K*_L_, was introduced to evaluate the cone index of the stabilized soils. It was discovered that the proposed equation unified the assessment of the improvement of the three Kumamoto clay samples in a unified manner owing to the new parameter *K*_L_.

## Figures and Tables

**Table 1:** Physical properties of Kumamoto clay.

Clay Type	Fine Particle Content *F*c * (%)	Liquid Limit *w*_L_ (%)	Plastic Limit *w*_p_ (%)	Plasticity Index *I*_p_	Ignition Loss LOI (%)	Density *ρ*_s_ (g/cm^3^)	Natural Water Content *w*_n_ (%)
Sample A	64.77	121.98	82.12	39.86	23.4	2.32	160
Sample B	65.05	142.00	93.68	48.32	19.2	2.27	152
Sample C	67.46	158.00	96.92	61.08	17.3	2.55	208

* Fine particle content *F*c: The maximum particle size is 0.075 mm.

**Table 2:** Compaction and cone penetration test conditions.

Clay	Kumamoto Clay (Sample A, Sample B, Sample C)
Type of solidifying agent	OPC and DF
Mold	Inner diameter was 100 mm, and the capacity was about 0.001 m^3^
Rammer	The weight of the heavy hammer was 2.5 kg, the falling height was 300 mm, and the falling method was free-fall
Solidification time	28 days (Cone index test after 28 days of mixing)
Cone penetrometer	Tip angle 30°, bottom area 320 mm^2^
Penetration rate	1 cm/s
Measuring method	The penetration resistance force at the distance of penetration of 50, 75, 100 mm was measured, and the value obtained by dividing the average value by the cone bottom area was defined as the cone index.
Product type of load measurement device	RZTA-1000 N produced by IMADA

**Table 3:** List of experimental programs.

Series	Sample	Water Content (%)	Content of Solidifying Agent (kg/m^3^)	Type of Solidifying Agent
1	A	160	100, 200, 300, 400	OPC, DF
	B	160	100, 200, 300, 400	OPC, DF
	C	200	100, 200, 300, 400	OPC, DF
2	A	100, 120, 140, 160	100	OPC, DF
	B	100, 115, 135, 160	100	OPC, DF
	C	120, 140, 160, 200	100	OPC, DF

**Table 4:** Parameters *A*, *B*, and d of Equation (4) for stabilized clay.

Sample	Solidifying Agent	*d*	*A* (kN/m^2^)	*B* (m^3^/kg)	*R* ^2^
Sample A	DF	3.3	7.52	0.045	0.90
	OPC	2.5	7.36	0.0506	0.92
Sample B	DF	3.0	5.33	0.0547	0.98
	OPC	2.3	7.97	0.0523	0.97
Sample C	DF	2.8	14.96	0.0703	0.92
	OPC	2.3	14.41	0.0713	0.91

**Table 5:** Parameters of *A*_L_, *B*_L_, and *d*_L_ in Equation (7) for stabilized clay.

Solidifying Agents	Sample Size	*d*_L_	*A*_L_ (kN/m^2^)	*B*_L_ (m^3^/kg)	Correlation Coefficient R^2^
DF	21	2.9	11.7	0.0178	0.82 *
OPC	18	2.3	16.71	0.0219	0.83 *

* Significant level: 5%.

## Nomenclature

*A*	Represents the intercept of Equation (4) on the *Y*-axis (kN/m^2^)
*A*_L_	Represents the intercept of Equation (7) on the *Y*-axis (kN/m^2^)
*A*_w_	The ratio of stabilization binder weight to the dry weight of clay
*B*	Represents the slope in Equation (4) (m^3^/kg)
*B*_L_	Represents the slope in Equation (7) (m^3^/kg)
*C*	Mixing content of solidifying agent (ordinary Portland cement and in manuscript) (kg/m^3^)
*d*	A coefficient representing the effect of the water content
*d*_L_	A coefficient representing the effect of the water content based on the liquid limit
*F*c	Fine particle content (particle size less than 0.075 mm) (%)
*I*_p_	Plasticity index
*K*	A parameter for describing solidifying agent content considering variation of water content (kg/m^3^)
*K*_L_	Modified content of the solidifying agent (kg/m^3^)
LOI	Ignition loss at 750 °C (%)
*N*_d_	The number of blows per penetration depth of 0.1 m (times/0.1 m)
*q*_c_	Cone index (kN/m^2^)
*q*_u_	Unconfined compressive strength (kN/m^2^)
*q*_u1_	Unconfined compressive strength of undisturbed clays (kN/m^2^)
*q*_u2_	Unconfined compressive strength of remolded clays (kN/m^2^)
R^2^	Correlation coefficient
*S*t	Sensitivity ratio
*w*	Water content (%)
*w*_L_	Liquid limit (%)
*w*_n_	Natural water content (%)
*w*_p_	Plastic limit (%)
ρs	Density of the soil particle (g/cm^3^)

## References

[1] MOE (Ministry of the Environment of Japan). https://www.env.go.jp/council/09water/y092-05/mat04-1.pdf.

[2] Sumi T. Selection of dam sediment database and appropriate reservoir sediment management measures in Japan. Proceedings of the ICOLD 2013 International Symposium.

[3] Satoh T., Tsuchida T., Mitsukuri K., Hong Z. (2001). Field placing test of lightweight treated soil under seawater in Kumamoto port. Soils Found..

[4] Satoh T. (2003). Application of pneumatic flow mixing method Central Japan International Airport construction. J. Jpn. Soc. Civ. Eng..

[5] Jamnongpipatkul P., Dechasakulsom M., Sukolrat J. Application of air-foam stabilized soil for bridge-embankment transition zone in Thailand. Proceedings of the GeoHuman International Conference 2009.

[6] Ifediniru C., Ekeocha N.E. (2022). Performance of cement-stabilized weak subgrade for highway embankment construction in Southeast Nigeria. Int. J. Geo-Eng..

[7] Chindaprasirt P., Homwuttiwong S., Sirivivatnanon V. (2004). Influence of fly ash fineness on strength, drying shrinkage and sulfate resistance of blended cement mortar. Cem. Concr. Res..

[8] Singh S.P., Tripathy D.P., Ranjith P.G. (2008). Performance evaluation of cement stabilized fly ash–GBFS mixes as a highway construction material. Waste Manag..

[9] Yoobanpot N., Jamsawang P., Horpibulsuk S. (2017). Strength behavior and microstructural characteristics of soft clay stabilized with cement kiln dust and fly ash residue. Appl. Clay Sci..

[10] Xiao D., Jiang G.L., Liao D., Hu Y.F., Liu X.F. (2018). Influence of cement-fly ash-gravel pile-supported approach embankment on abutment piles in soft ground. J. Rock Mech. Geotech. Eng..

[11] Horpibulsuk S., Rachan R., Raksachon Y. (2009). Role of Fly Ash on Strength and Microstructure Development in Blended Cement Stabilized Silty Clay. Soils Found..

[12] Papadakis V.G. (2000). Effect of fly ash on Portland cement systems Part II. High-calcium fly ash. Cem. Concr. Res..

[13] Tang Y.X., Miyazaki Y., Tsuchida T. (2001). Practice of reused dredgings by cement treatment. Soils Found..

[14] Miura N., Horpibulsuk S., Nagaraj T.S. (2001). Engineering behavior of cement stabilized clay at high water content. Soils Found..

[15] Abrams D.A. (1919). Effect of Fineness of Cement: By Duff A. Abrams.

[16] Horpibulsuk S., Miura N., Nagaraj T.S. (2005). Clay–water/cement ratio identity of cement admixed soft clay. Geotech. Geoenvironmental Eng..

[17] Sakka H., Ochiai H., Yasufuku N., Omine K. (2002). Evaluation of deformation-strength properties of cement-stabilized soils by falling weight deformation measurement apparatus. J. Jpn. Soc. Civ. Eng..

[18] Moreira E.B., Baldovino J.A., Rose J.L., Izzo R.L.S. (2019). Effects of porosity, dry unit weight, cement content and void/cement ratio on unconfined compressive strength of roof tile waste-silty soil mixtures. J. Rock Mech. Geotech. Eng..

[19] Mitsui T., Yoshikawa T., Ikeda A., Aoyama K., Nakagawa K. (2001). A Basic Study of Improved Soils with Different Fine Grain Contents by Laboratory Mixing Tests. J. Jpn. Soc. Civ. Eng..

[20] Hussan A., Levacher D., Mezazigh S., Jardin L. (2022). Valorization of a Highly Organic Sediment: From Conventional Binders to a Geopolymer Approach. J. Compos. Sci..

[21] (2018). Method for Dynamic Cone Penetration Test.

[22] (2020). Test Method for Ignition Loss of Soils.

[23] (2020). Test Method for Particle Size Distribution of Soils.

[24] (2020). Test Method for Liquid Limit and Plastic Limit of Soils.

[25] (2020). Method for Unconfined Compression Test of Soils.

[26] (2020). Test Method for Water Content of Soils.

[27] (2020). Practice for Making and Curing Compacted Stabilized Soil Specimens Using a Rammer.

[28] (2020). Test method for Soil Compaction Using a Rammer.

[29] (2020). Test Method for Cone Index of Compacted Soils.

[30] Santisteban J.I., Mediavilla R., Lopez-Pamo E., Dabrio C.J., Zapata M., Garcia M., Castano S., Martínez-Alfaro P.E. (2004). Loss on ignition: A qualitative or quantitative method for organic matter and carbonate mineral content in sediments. J. Paleolimnol..

[31] Nielsen S., Stefanakis A.I. (2020). Sustainable dewatering of industrial sludges in sludge treatment reed beds: Experiences from pilot and full-scale studies under different climates. Appl. Sci..

[32] Aitkenhead M.J., Donnelly D., Sutherland L., Miller D.G., Coull M.C., Black H.I.J. (2015). Predicting Scottish topsoil organic matter content from colour and environmental factors. Eur. J. Soil Sci..

[33] Ghosh A., Subbarao C. (2007). Strength Characteristics of Class F Fly Ash Modified with Lime and Gypsum. Geotech. Geoenvironmental Eng..

[34] Horpibulsuk S., Liu M.D., Liyanapathirana D.S., Suebsuk J. (2010). Behavior of cemented clay simulated via the theoretical framework of the Structured Cam Clay model. Comput. Geotech..

[35] Wang D.X., Edine A.N., Rachid Z. (2013). Strength and deformation properties of Dunkirk marine sediments solidified with cement, lime and fly ash. Eng. Geol..

[36] Furlan A.P., Razakamanantsoa A., Ranaivomanana H., Amiri O., Levacher D., Deneele D. (2021). Effect of Fly Ash on microstructural and resistance characteristics of dredged sediment stabilized with lime and cement. Constr. Build. Mater..

[37] Silitonga E., Levacher D., Mezazigh S. (2010). Utilization of fly ash for stabilization of marine dredged sediment. Eur. J. Environ. Civ. Eng..

[38] Horpibulsuk S., Katkan W., Sirilerdwattana W., Rachan R. (2006). Strength Development in Cement Stabilized Low Plasticity and Coarse Grained Soils: Laboratory and Field Study. Soils Found..

[39] Horpibulsuk S., Miura N., Nagaraj T.S. (2003). Assessment of strength development in cement-admixed high water content clays with Abrams’ law as a basis. Geotechnique.

[40] Horpibulsuk S., Rachan R., Suddeepong A., Chinkulkijniwat A. (2011). Strength development in cement admixed Bangkok clay: Laboratory and field investigations. Soils Found..

[41] Lorenzo G.A., Bergado D.T. (2004). Fundamental parameters of cement-admixed clay—New approach. Geotech. Geoenvironmental Eng..

[42] Jongpradist P., Jumlongrach N., Youwai S., Chucheepsakul S. (2010). Influence of Fly Ash on Unconfined Compressive Strength of Cement-Admixed Clay at High Water Content. J. Mater. Civ. Eng..

[43] Uchida K., Fukubayashi Y., Yamashita J. (1985). Effect of Humic Acid in Soil on Hydration of Irwin Cement. Annu. Rep. Cem. Concr. Eng..

[44] Okabayashi S., Omori H., Yanagihara H., Takahashi S. (2004). Principles and applications of soil hardening: 2. Cement and cement-based solidifying material chemistry. Soil Mech. Found. Eng..

[45] Horpibulsuk S., Yangsukkaseam N., Chinkulkijniwat A., Du Y.J. (2011). Compressibility and permeability of Bangkok clay compared with kaolinite and bentonite. Appl. Clay Sci..

[46] Flemmy S.O., Omine K., Zhang Z. (2020). Effect of Installed Geotextile/Polyester and Biodegradable Materials for Dewatering Soft Clay. Advances in Sustainable Construction and Resource Management.

[47] Flemmy S.O., Omine K., Zhang Z. (2019). Soft clay improvement technique by dewatering and dewatering sandy soil. Int. J. Geomate.

